# Supplementary material for: Examining perinatal regionalization in practice: a network analysis of maternal transport in Georgia
Source: BMC Health Serv Res. 2025 Jul 1;25:862. doi: 10.1186/s12913-025-13025-9 (PMC12220406; doi:10.1186/s12913-025-13025-9)
Supplement: Supplementary file 1 — Supplementary Material 1. [file 12913_2025_13025_MOESM1_ESM.docx]

# **Appendix A: Additional Methods**

### **A1. Data inclusion criteria**


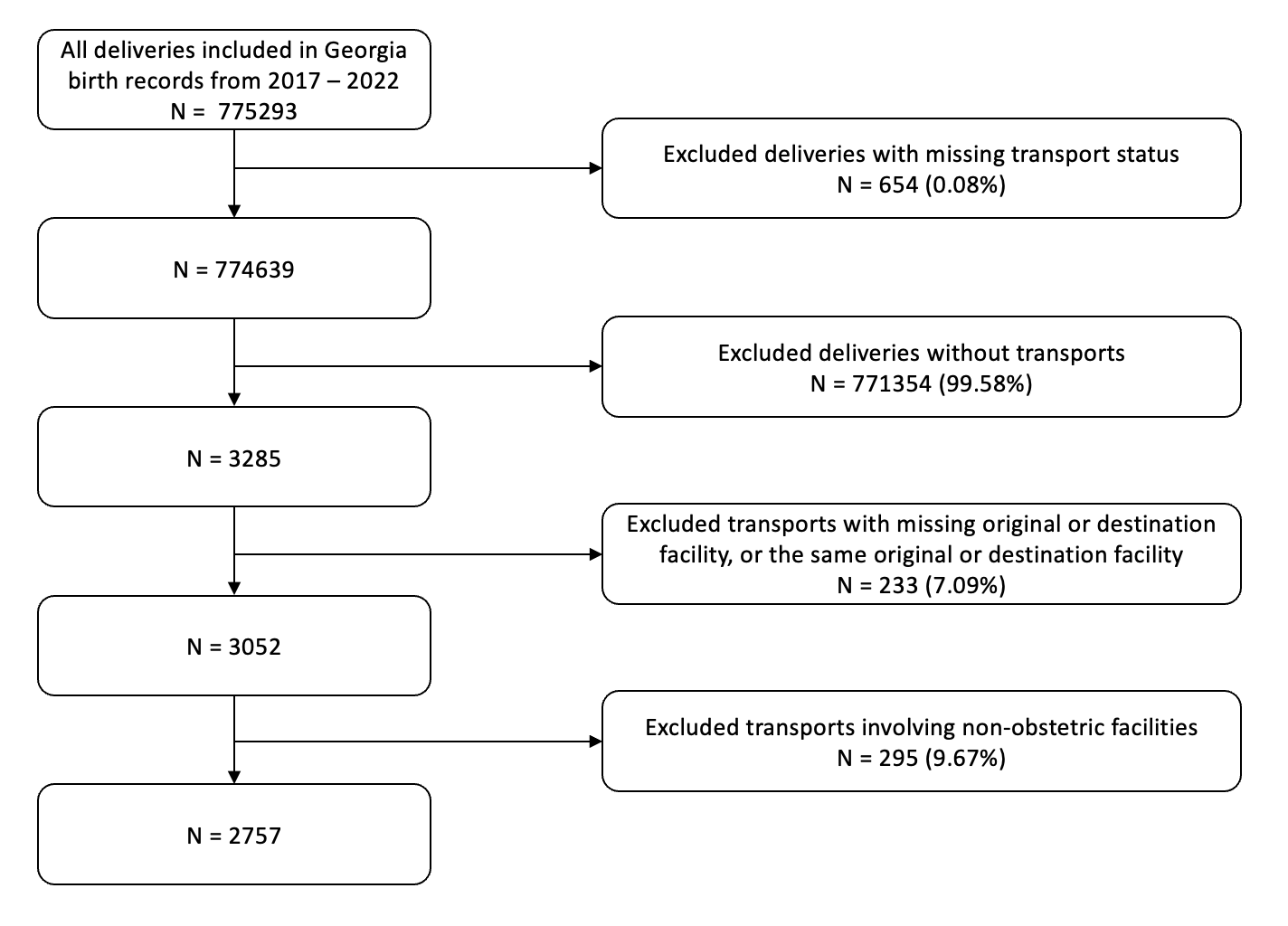


**Appendix Figure A1. Data inclusion criteria for the study sample.** To construct maternal transport networks among obstetric facilities, we excluded deliveries that had missing transport status, did not involve a transport, had missing records of original or destination facility, had the same original or destination facility, or involved non-obstetric facilities.

### **A2. Description of variables**

**Appendix Table A2. Description of variables analyzed in the study.**

| Variable name | Description | Type | Values |
| --- | --- | --- | --- |
| Age in years | The mother's age in years at the time of delivery. | Numeric | range 10-55 years |
| Race | White = a person having origins in any of the original peoples of Europe, the Middle East or North Africa; Black or African-American = A person having origins in any of the black racial groups of Africa; Asian=A person having origins in any of the original peoples of the Far East, Southeast Asia, or the Indian subcontinent including, for example, Cambodia, China, India, Japan, Korea, Malaysia, Pakistan, the Philippine Islands, Thailand and Vietnam; American Indian/Alaska Native=A person having origins in any of the original peoples of North and South America (including Central American), and who maintains tribal affiliation or community attachment; Native Hawaiian or Other Pacific Islander=A person having origins in any of the original peoples of Hawaii, Guam, Samoa, or other Pacific Islands. Multiracial = 2 or more of these races (OMB-15, 1997). | Categorical | White, Black or African-American, Asian, American Indian/Alaska Native, Native Hawaiian or Other Pacific Islander, Multiracial |
| Ethnicity | Ethnicity, currently limited to asking whether the person is “Hispanic or Latino” (A person of Mexican, Puerto Rican, Cuban, South or Central American, or other Spanish culture or origin, regardless of race) (OMB-15, 1997). | Categorical | Hispanic, Non-Hispanic |
| Location of residence | Patients’ residence by linking 2020 census block group to the Rural-Urban Continuum Codes (RUCCs): urban (RUCCs of 1-3), suburban (RUCCs of 4-6), and rural (RUCCs of 7-9). | Categorical | Urban, Suburban, Rural |
| Education level | The last grade of formal education completed. | Categorical | Less than 9th Grade, 9th through 11th Grade, High School Diploma or GED (12), Some College or Higher |
| Payor | Principal source of payment | Categorical | Commercial Insurance, Medicaid, Medicaid Applicants, or Medicaid Managed Care, Other Insurance, Government Assistance, or Other Non-specified Managed Care, Self-Pay, TRICARE |
| APNCU index | The Adequacy of Prenatal Care Utilization Index | Categorical | Inadequate, Intermediate, Adequate, Adequate plus |
| Tobacco use | Whether patient used tobacco products during this pregnancy. | Categorical | Yes, No |
| Number of medical risk factors | Number of medical risk factors patient has among: pre-pregnancy diabetes, gestational diabetes, eclampsia, pre-pregnancy hypertension, gestational/pregnancy-associated hypertension, previous preterm birth, and previous Cesarean delivery. | Numeric | range 0-7 |
| Gestation weeks | The standard calculation of number of completed weeks of gestation based on Obstetric Estimate. | Numeric | range 20-44 completed weeks inclusive |
| Plurality | Number of fetuses for this pregnancy. | Numeric | range 1-8 |
| Fetal presentation | The fetal presentation during delivery of the fetus or live birth. | Categorical | Cephalic, Breech, Other |
| Final method of delivery | Final route and method of delivery. | Categorical | Vaginal, Cesarean |
| Infant transfer after birth | Whether the Infant was transferred to another facility after birth | Categorical | Yes, No |
| Abnormal condition relating to NICU admission | Abnormal condition relating to infant - NICU admission. | Categorical | Yes, No |
| Received WIC for food | Whether patient received WIC (Women Infants & Children) services any time during pregnancy. | Categorical | Yes, No |
| Received steroid for fetal lung maturation prior to delivery | Whether patient received steroids (glucocorticoids) for fetal lung maturation prior to delivery. | Categorical | Yes, No |
| Maternal transfusion | Whether patient has Maternal Morbidity - Maternal transfusion. | Categorical | Yes, No |
| Origin LOC | Level of Care of the origin facility | Categorical | Birth center, level I, level II, Level III, RPC |
| Destination LOC | Level of Care of the destination facility | Categorical | level II, Level III, RPC |
| Same system | Whether origin facility and destination facility belong to the same healthcare system. | Categorical | Yes, No |

### **A3. Representation of maternal transport networks**


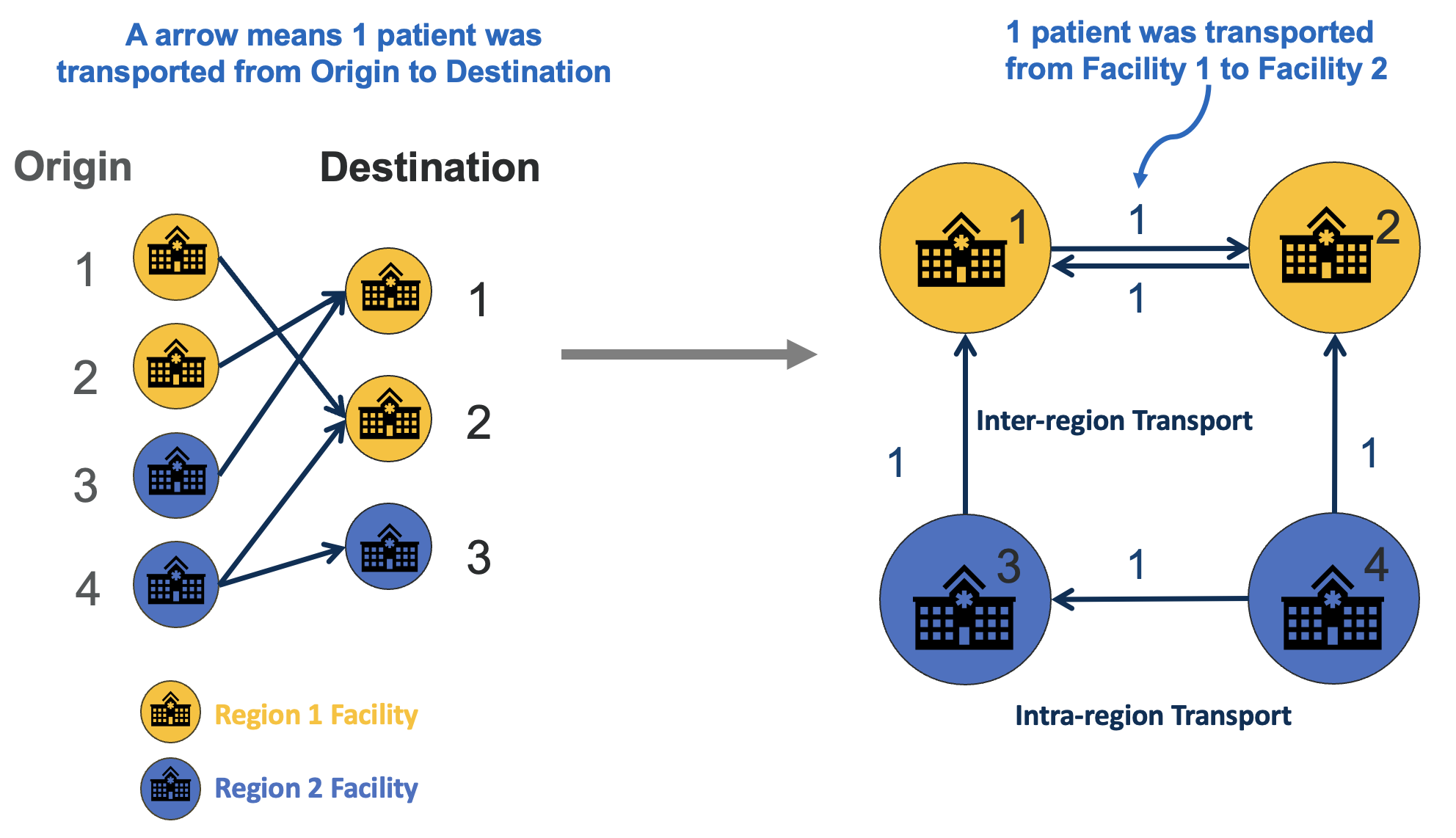


**Appendix Figure A3. Illustration of the network representation of maternal transport routes.** Each node (circle) indicates a facility with its color representing its designated perinatal region. Arrows represent that a transport occurred between the indicated origin facility and the destination facility.

### **A4. Characterization of the intra-region maternal transport**

To understand the network structure within each region, we performed intra-region network analysis by constructing “sub-networks” for each DPR. Specifically, for each DPR, we used only facilities (nodes) that belong to the given DPR and only transports among those facilities (i.e., edges that connect these nodes). We characterized the full observed networks and each DPR sub-network using several common network metrics that describe different aspects of network structure and strength. For each network, we summarized number of nodes and edges. We also calculated the following network metrics: Katz centrality,^1,2^ density,^3^ efficiency,^4^ resilience,^5^ and clustering coefficient.^6^ Comparing sub-network metrics with global metrics of the full network can identify how each sub-network contributes to the overall network structure and dynamics.

#### **Network Metrics**

*Katz centrality* measures the influence of a node to all other nodes in the network. Facilities with high value of Katz centrality are crucial nodes in the network, as they not only handle a large number of maternal transports but also maintain connections with other important facilities. It is useful in detecting the “hub and spoke” pattern in the network. Nodes with high Katz centrality can be considered central hubs in the hub-and-spoke pattern, as they are influential to other nodes. DPRs with high values of average Katz centrality over RPCs and efficiency are better equipped to coordinate patient care, share critical medical information promptly, and optimize resource allocation with other providers from the same region.

*Density* measures the proportion of actual connections to possible connections in the network. High density may indicate a well-integrated network, while low density may suggest fragmentation. A subnetwork with high density may indicate a tightly integrated group of facilities that frequently collaborate on maternal transport.

*Efficiency* is calculated using the multiplicative inverse of the shortest path length between nodes. In a maternal transport network, higher efficiency indicates that pregnant people can be quickly transported to a risk-appropriate facility.

*Resilience* measures how the network reacts to node or edge removal. It is calculated by simulating node removal from the network and measuring the impact on the size of the largest connected component. Subnetworks with high resilience value are robust to the removal of a facility.

*Clustering coefficient* measures the extent to which nodes in a network tend to cluster together. This can indicate the presence of communities or groups or facilities who transport patients more frequently among themselves than with facilities outside their regions.

### **A5. Comparison of observed networks and state guidelines for regionalization**

To identify algorithm-detected communities, we applied the *Louvain community detection algorithm*, which involves a two-phase modularity optimization process and works well with both undirectional and directional network graphs.^7,8^ In our analysis, we let the algorithm identify the number of communities by detecting community structures that yield the maximum modularity gain of the network, rather than pre-specified the desired number of communities. We quantified the degree of concordance between the algorithm-detected communities and the state’s defined DPRs using a χ2-test.

# **Appendix B: Additional Results**

### **B1. Representation of maternal transport networks**

**Appendix Table B1. Maternal transport by levels of care.**

|  | ***Destination*** | | | |
| --- | --- | --- | --- | --- |
|  | Level I | Level II | Level III | RPC |
| ***Origin*** |  |  |  |  |
| Birth Center | 0 | 0 | 223 (79.6%) | 57 (20.4%) |
| Level I | 12 (1.4%) | 17 (2.0%) | 59 (7.0%) | 756 (89.6%) |
| Level II | 4 (0.0%) | 30 (2.1%) | 41 (2.9%) | 1411 (95.0%) |
| Level III | 9 (9.0%) | 7 (7.0%) | 19 (19.0%) | 65 (65.0%) |
| RPC | 8 (17.0%) | 6 (12.8%) | 19 (40.4%) | 14 (29.8%) |
| Abbreviations: RPC, regional perinatal center. | | | | |

### **B2. Characterization of the inter-region maternal transport**

**Appendix Table B2.1. Nodes, edges, and transport volume across DPRs.**

|  | **inter-region nodes**^a^  **(n%)** | **inter-region Edges**^b^  **(n%)** | **inter-region transports**^c^ **(n%)** | **Inter-region transports per edge**^d^ |
| --- | --- | --- | --- | --- |
| **Full transport network** | 52/80 (65.00) | 80/199 (40.20) | 468/2757 (16.97) | 5.85 |
| ***DPRs*** |  |  |  |  |
| **Albany** | 6/7 (85.71) | 25/32 (78.13) | 259/619 (41.84) | 10.36 |
| **Atlanta** | 17/34 (50.00) | 29/87 (33.33) | 55/428 (12.85) | 1.90 |
| **Augusta** | 4/7 (57.14) | 22/30 (73.33) | 60/164 (36.59) | 2.73 |
| **Columbus** | 6/9 (66.67) | 25/34 (73.53) | 155/413 (37.53) | 6.20 |
| **Macon** | 9/10 (90.00) | 38/53 (71.70) | 337/864 (39.00) | 8.87 |
| **Savannah** | 10/13 (76.92) | 21/43 (48.84) | 70/737 (9.50) | 3.33 |
| Abbreviations: DPR, designated perinatal region.   1. Inter-region nodes represent the number of nodes that have a connecting edge with at least one node from another DRP. 2. For each DPR, inter-region edges represent the number of edges that are connecting between a facility from the DPR and another facility from a different DPR. 3. Inter-region transports represent the number of transports through inter-region edges. 4. Inter-region transports per edge are calculated by dividing inter-region transports by the number of inter-region edges. | | | | |

**Table B2.2. Maternal transport by origin and destination DPRs.**

|  | ***Destination DPR*** | | | | | |
| --- | --- | --- | --- | --- | --- | --- |
|  | Albany | Atlanta | Augusta | Columbus | Macon | Savannah |
| ***Origin DPR*** |  |  |  |  |  |  |
| Albany | 360 | 2 | 6 | 7 | 11 | 2 |
| Atlanta | 0 | 373 | 6^a^ | 6 | 9 | 1 |
| Augusta | 0 | 5 | 104 | 0 | 0 | 3 |
| Columbus | 41 | 22 | 0 | 258 | 52 | 0 |
| Macon | 185 | 0 | 20 | 26 | 527 | 24 |
| Savannah | 5 | 4 | 20 | 1 | 10 | 667 |
| Abbreviations: DPR, designated perinatal region.   1. Grey shading indicates neighboring regions. Number of inter-region transports across neighboring DPRs were 454 out of 468 (97%) inter-region transports. | | | | | | |

### **B3. Characterization of the intra-region maternal transport**

**Appendix Table B3. Descriptive metrics of intra-region maternal transport networks.**

|  | **Nodes** | **Edges**^a^ | | **Number of patients** | **Katz centrality**^b^ | **Density** | **Efficiency**^c^ | **Resilience**^d^ | **Clustering coefficient**^e^ | |
| --- | --- | --- | --- | --- | --- | --- | --- | --- | --- | --- |
| **Full transport network** | 80 | 199 | 2757 | | 0.1280 | 0.0315 | 0.3918 | 0.9853 | 0.1910 | |
| ***Sub-networks by DPRs*** |  |  |  | |  |  |  |  |  |  |
| **Albany** | 7 | 7 | 360 | | 0.4526 | 0.1667 | 0.6429 | 0.7551 | 0.0000^e^ | |
| **Atlanta** | 34 | 58 | 373 | | 0.1691 | 0.0517 | 0.3553 | 0.9632 | 0.1555 | |
| **Augusta** | 7 | 8 | 104 | | 0.4087 | 0.1905 | 0.6667 | 0.7755 | 0.2524 | |
| **Columbus** | 9 | 9 | 258 | | 0.3851 | 0.1250 | 0.6250 | 0.8148 | 0.1131 | |
| **Macon** | 10 | 15 | 527 | | 0.3973 | 0.1667 | 0.6333 | 0.8400 | 0.2357 | |
| **Savannah** | 13 | 22 | 667 | | 0.4272 | 0.1410 | 0.6154 | 0.8876 | 0.3455 | |
| Abbreviations: DPR, designated perinatal region.   1. Edge weights between two nodes are normalized to account for different transport volume in each DPR in the calculation of Katz centrality and clustering coefficient. We calculated the “adjusted weight” of an edge by dividing the number of transports on the corresponding edge by the maximum volume at both the origin and the destination facility, and taking the maximum of these two to reflect a strong connection strength between two facilities if at least one out of the facility pairs transports a significant fraction of patients from or to another facility. 2. Katz centrality, resilience, and clustering coefficient are node-specific metrics. We reported the average Katz centrality over the Regional Perinatal Centers (RPCs) in each DPR and average resilience and clustering coefficient over all nodes in each DPR. 3. Efficiency is measured on node pairs using undirected graphs. We reported the average efficiency over all node pairs in each region. 4. Efficiency and resilience were calculated using undirected graphs as they are defined for those. All other metrics were calculated using directional graphs. 5. Clustering coefficient of a node is defined as zero if a node has less than two adjacent edges. Zero clustering coefficient of the Albany DPR suggests every node in this DPR has only one edge. | | | | | | | | | | |

Basic network metrics including number of nodes and edges, percentage of transports between two facilities in the same DPR, and metrics to measure different aspects of network structure and strength are shown in **Appendix Table B3.** We used Katz centrality, density, efficiency, resilience, and clustering coefficient to assess the additional architecture of maternal transport full network and subnetworks. Each DPR exhibits different regionalization patterns. Atlanta DPR had the highest number of facilities and a moderate number of transports. In contrast, Albany and Augusta DPRs showed lower activity in terms of transport volume, particularly in same-region maternal transports. Measures of Katz centrality and efficiency show that RPCs were the most influential facilities within their respective DPR subnetworks. However, in the Atlanta DPR, low RPC Katz centrality and efficiency values suggested a fragmented structure. This fragmentation was also confirmed by the division of the Atlanta DPR into three algorithm-detected communities. Specifically, when looking at rank of the Katz centrality of the RPCs by region, we noticed that RPCs in all other DPRs except Atlanta DPR were the 1^st^ ranked facilities in their DPRs. RPCs in Atlanta, however, were ranked 4^th^, 10^th^, 29^th^ and 30^th^ respectively, with the highest ranked RPC and the lowest ranked two RPCs in Atlanta DPR being neonatal RPCs. We also noticed that Augusta DPR had the highest value of density, in addition to a high value of efficiency. This suggests that maternal transport routes in Augusta DPR are interconnected over different facilities in this region. We noticed that Albany DPR is the least resilient among all regions, followed by Augusta, meaning that care coordination on maternal transport among those regions can be more easily impacted by the shutdown of obstetric units. We also observed that clustering coefficient in the Albany DPR is zero, which suggests that each facility in this DPR has a fixed maternal transport route. A subnetwork with a high clustering coefficient such as Savannah DPR may represent a group of providers who frequently collaborate on maternal transport.

1. Katz L. A new status index derived from sociometric analysis. *Psychometrika*. 1953;18(1):39-43. doi:10.1007/BF02289026

2. Newman M, ed. In: *Networks: An Introduction*. 1st ed. Oxford University Press; 2010:720. doi:10.1093/acprof:oso/9780199206650.002.0003

3. de Laat M, Lally V, Lipponen L, Simons RJ. Investigating patterns of interaction in networked learning and computer-supported collaborative learning: A role for Social Network Analysis. *Int J Comput-Support Collab Learn*. 2007;2(1):87-103. doi:10.1007/s11412-007-9006-4

4. Latora V, Marchiori M. Efficient Behavior of Small-World Networks. *Phys Rev Lett*. 2001;87(19):198701. doi:10.1103/PhysRevLett.87.198701

5. Albert R, Jeong H, Barabási AL. Error and attack tolerance of complex networks. *Nature*. 2000;406(6794):378-382. doi:10.1038/35019019

6. Onnela JP, Saramäki J, Kertész J, Kaski K. Intensity and coherence of motifs in weighted complex networks. *Phys Rev E Stat Nonlin Soft Matter Phys*. 2005;71(6 Pt 2):065103. doi:10.1103/PhysRevE.71.065103

7. Blondel VD, Guillaume JL, Lambiotte R, Lefebvre E. Fast unfolding of communities in large networks. *J Stat Mech Theory Exp*. 2008;2008(10):P10008. doi:10.1088/1742-5468/2008/10/P10008

8. Dugué N, Perez A. *Directed Louvain : Maximizing Modularity in Directed Networks*. Université d’Orléans; 2015. Accessed September 6, 2024. https://hal.science/hal-01231784
